# Euro-Asian hybrids of *Echinococcus multilocularis* from red foxes in northern and northeastern Poland result from secondary contact between long-isolated populations

**DOI:** 10.1038/s41598-026-40313-z

**Published:** 2026-02-20

**Authors:** Paweł Gładysz, Dorota Bielińska-Wąż, Piotr Wąż, Małgorzata Samorek-Pieróg, Jacek Karamon, Anna Lass

**Affiliations:** 1 Division of Tropical Parasitology, Department of Tropical Medicine and Parasitology, Institute of Maritime and Tropical Medicine, Medical University of Gdańsk, Powstania Styczniowego 9B, 81-519 Gdynia, Poland; 2 Department of Radiological Informatics and Statistics, Medical University of Gdańsk, 80-210 Gdańsk, Poland; 3 Department of Nuclear Medicine, Medical University of Gdańsk, 80-210 Gdańsk, Poland; 4 National Veterinary Research Institute, Partyzantów 57, 24-100 Puławy, Poland

**Keywords:** Alignment-free method, Alveolar echinococcosis, Asian origin, Phylogeography, Ecology, Ecology, Evolution, Genetics, Microbiology, Molecular biology

## Abstract

We investigated the mitochondrial genetic diversity of *Echinococcus multilocularis* in red foxes from northern and northeastern Poland, and tested the hypothesis that the presence of Asian haplotypes in Poland stems from their introduction from Northeast Asia to Eastern Europe. We targeted the cytochrome c oxidase subunit 1 gene (*cox1*) of 263 tapeworms from 59 red foxes hunted in selected districts of Pomorskie, Warmińsko-Mazurskie, and Podlaskie Voivodships. We calculated several measures of genetic variation, and constructed median-joining haplotype networks and a RelTime timetree. In addition, a novel alignment-free computational method was applied: the 4D-Dynamic Representation of DNA/RNA Sequences combined with *K*-means clustering. PCR and sequencing were successful for 252 tapeworms. We identified six haplotypes, three of which were novel. The mismatch distribution was bimodal. The fixation index (F_ST_) between the European and Asian haplogroups was 0.97. Our findings suggest secondary contact between two previously isolated metapopulations with distinct genetic lineages. Clustering patterns appear to support the hypothesis that Asian variants of *E. multilocularis* spread into northeastern Poland as a consequence of multiple introductions of raccoon dogs from the Soviet Far East to Eastern Europe.

**Supplementary Information:**

The online version contains supplementary material available at 10.1038/s41598-026-40313-z.

## Introduction

Research into the phylogeography of parasites and their hosts offers insight into past modes of pathogen dispersal, possible present mechanisms at play, and potential directions of future spread. *Echinococcus multilocularis* (Leuckart, 1863) is the causative agent of alveolar echinococcosis, a severe zoonotic disease with a long incubation period and poor prognosis unless treated^1,2^. The phylogeography of the species in Europe is shaped primarily by the refugial and post-glacial history of the red fox and the recession of the Arctic fox^3–7^.

*Echinococcus multilocularis* gene flow to a large extent depends on chance. The parasite has little agency: its movement relies on the migratory behaviour of its hosts and passive egg transport. When mating, it remains limited to conspecifics in the definitive host’s small intestine, most of which likely stem from a single metacestode—making geitonogamy and autogamy its predominant modes of sexual reproduction^8,9^. It comes as no surprise, then, that detection of autochthonous extra-continental variants and cross-continental hybrids raises questions about their origin as well as their pathogenicity and virulence for local host communities, including people^10^.

To this day, Asian or part-Asian adult tapeworms of *E. multilocularis* west of the Urals have been observed in definitive hosts from European Russia^11^, Poland^12^, and Latvia^5^. A local Asian mitochondrial variant was first discovered in Poland in 2017^13^. Concatenated sequences of three mitochondrial markers revealed a haplotype clustering away from European samples and close to Japanese and Kazakh ones in a statistical parsimony network. Of 74 tapeworms collected across the country, seven (9.5%) from northeastern and central Poland belonged to the foreign haplotype.

A follow-up study using the microsatellite EmsB and the complete cytochrome c oxidase subunit 1 gene (*cox1*) revealed introgression^12^. Thirty-two out of 349 Polish tapeworms (9.2%) were Asian by descent in the nuclear and/or mitochondrial genome, extending as far west as the Polish-German border. Seventeen of them (4.9%) were Euro-Asian hybrids. Later investigations demonstrated cases of Asian variants parasitising pigs ^14^ and people ^15^, indicating that their eggs contaminate soil and produce.

A few explanations for the presence of Asian genomic elements in the Polish *E. multilocularis* population have been proposed: (a) recent introduction by migrating canids^12,13^; (b) human-mediated introduction with raccoon dogs transported in the 20^th^ century from Northeast Asia to Eastern Europe^5^; and (c) ancient polymorphism present for a long time: in this scenario, hybrids result from prolonged sympatry of European and Asian populations, sufficient to allow introgression, which, in turn, would indicate the existence of a westward gradient of progressively decreasing Asian contribution^12^.

In this study, we explored the *cox1* diversity of *E. multilocularis* from red foxes in northern and northeastern Poland. We tested the hypothesis that the presence of Asian haplotypes in Poland is a result of their introduction from Northeast Asia to Eastern Europe. We also compared our results with previously acquired nuclear data ^16^.

## Materials and methods

**Fig. 1:**
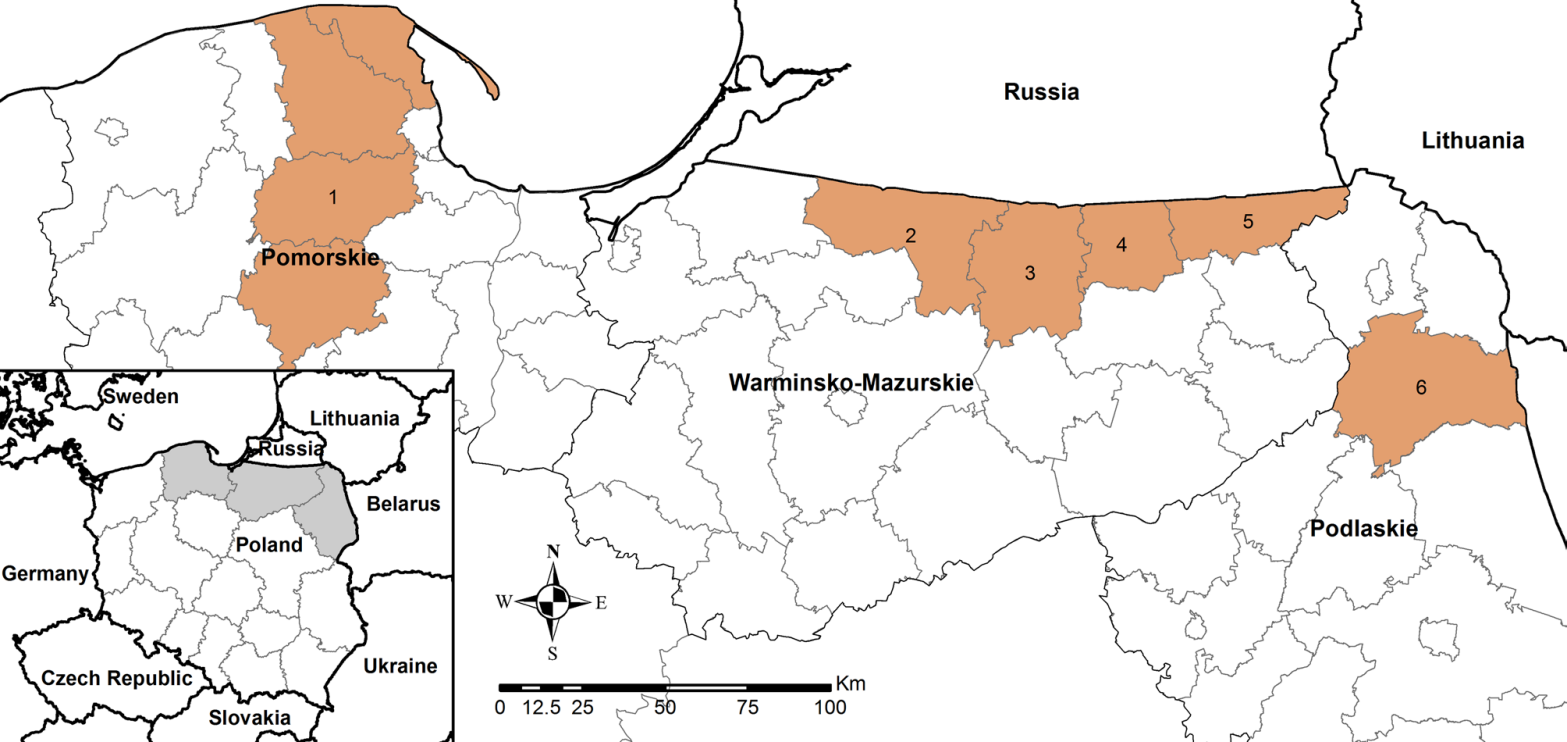
Map of the study area. Districts where red foxes were hunted are shown in orange: 1 – Puck, Wejherowo, Kartuzy, Kościerzyna; 2 – Bartoszyce; 3 – Kętrzyn; 4 – Węgorzewo; 5 – Gołdap; 6 – Augustów.

Two hundred and sixty-three tapeworms were removed from the intestines of 59 red foxes using the sedimentation and counting technique^17^. The foxes were hunted between 2022 and 2024 in Pomorskie Voivodship (Puck, Wejherowo, Kartuzy, and Kościerzyna Districts, treated as one area), Warmińsko-Mazurskie Voivodship (Bartoszyce, Gołdap, Kętrzyn, and Węgorzewo Districts), and Podlaskie Voivodship (Augustów District) (Figure 1). The foxes were obtained legally in collaboration with the Polish Hunting Association and the General Directorate of State Forests. The research project and all experimental protocols received approval from the Medical University of Gdańsk Bioethics Committee for Scientific Research (decision no. NKBBN/467/2021). All methods were carried out in accordance with relevant guidelines and regulations. For sampling details, see^18^ and^16^. Tapeworms were washed in physiological saline. Total genomic DNA was extracted from whole specimens using DNeasy Blood & Tissue Kit (Qiagen, Hilden, Germany, cat. no. 69506), following the manufacturer’s protocol.

### PCR amplification and sequencing of the complete *cox1* gene

We processed up to five randomly selected tapeworms per red fox: the same specimens as in^16^. PCRs to amplify the complete coding sequence of the cytochrome c oxidase subunit 1 gene (1,608 bp) were performed in a 25-μl reaction mixture containing 10–12 ng of template DNA, 12.5 μl of DreamTaq™ Hot Start Green PCR Master Mix (Thermo Scientific™, cat. no. K9021), 0.4 μM of primers EmcF and EmcR^19^, and 0.75 μl of DMSO (final concentration 3% v/v). PCR conditions were as follows: 95 °C for 3 min; 35 cycles of 95 °C for 30 s, 56.5 °C for 30 s, 72 °C for 1.5 min; and 72 °C for 15 min.

The selected marker is notorious for the presence of a poly-T region (positions 207–218), which causes polymerase slippage that leads to read corruption during sequencing^5,14^. To overcome this issue, we designed the reverse sequencing primer 10068R (5’-CCGTCTTCACATCCAACCCA-3’), which binds mid-gene and facilitates assembly of the complete coding sequence.

Amplicons were purified with Clean-Up Concentrator (A&A Biotechnology, Gdańsk, Poland, cat. no. 021-250C). Bidirectional sequencing was outsourced to Macrogen Europe (Amsterdam). Regions on both ends with more than a 1% chance of an error per base were automatically trimmed in Geneious Prime 2025.0.3 (https://www.geneious.com/). Forward and reverse strands were assembled into consensus sequences and checked for internal stop codons to ensure that orthologs were amplified. Species identity was confirmed with BLAST (https://blast.ncbi.nlm.nih.gov/Blast.cgi).

### Population genetic analysis

The following measures of genetic variation were calculated for the sampled regions: number of segregating sites (S), number of haplotypes (h), haplotype diversity (H_d_; the probability that two randomly chosen haplotypes are different^20^), average number of nucleotide differences between two randomly chosen sequences (K), and nucleotide diversity (Pi; the average number of nucleotide differences per site between two randomly chosen sequences^21^). Haplotypes were defined when at least one mutation occurred between two samples.

To quantify genetic differentiation, the fixation index (F_ST_) was calculated between two haplogroups, European and Asian. To assess whether the observed genetic differentiation was greater than expected by chance under panmixia, haplogroup labels were shuffled among sequences 1,000 times, and genetic differentiation statistics (H_S_/H_ST_, K_S_/K_ST_, K_S_*/K_ST_*, Z, Z*, and S_nn_) were calculated for each permutation and compared with their observed values. All calculations were done in DnaSP 6.12.03^22^.

A histogram of nucleotide site differences between pairs of individuals (mismatch distribution) was generated using the packages ‘adegenet’^23,24^ and ‘pegas’^25^ in R version 4.3.2^26^.

Two median-joining (MJ) haplotype networks (ε = 0)^27^ were created in PopART (https://popart.maths.otago.ac.nz/) and graphically refined in Inkscape (https://inkscape.org/about/): one for the PCR dataset only, and one for the PCR dataset combined with complete *cox1* sequences of Asian descent and records from North America and the Arctic deposited in GenBank (Supplementary File 1).

### Timetree

To estimate the divergence time of the European and Asian haplogroups in Poland, a timetree was constructed using MEGA12 version 12.0.9^28^. First, haplotypes observed in the PCR dataset were combined with other haplotypes detected in Poland^13,14^ and complete coding sequences of the *cox1* gene from *Echinococcus* species other than *E. multilocularis*, with *Taenia solium* serving as an outgroup^29^. All sequences were aligned using MUSCLE by translating codons to amino acids, performing the alignment, and then replacing the amino acids with the original codons.

The best nucleotide substitution model for constructing a Maximum Likelihood (ML) tree was determined using the ML statistical method and selected based on the Akaike Information Criterion and Bayesian Information Criterion. The robustness of the tree was tested by bootstrapping with 1000 replicates.

The topology of the ML tree was used to estimate a timetree by employing the RelTime method. Molecular dating was calibrated using the species pair *Echinococcus oligarthrus* and *Echinococcus vogeli*. The time to the most recent common ancestor (TMRCA) of these species was modelled as a normal distribution centred at 3.0 million years ago with a standard deviation of 0.3 million years (95% confidence interval: 2.41–3.59 million years), as in^30^.

This divergence time calibration constraint corresponds to the Great American Biotic Interchange, an event during which canid and felid ancestors of the modern endemic *E. oligarthrus* and *E. vogeli* definitive hosts migrated from North America to South America, following the formation of the Panamanian land bridge^29,31^.

### Comparison with nuclear data

In our previous article, we investigated the same set of 263 adult tapeworms using a nuclear marker, the microsatellite EmsB^16^. The study aimed to explore the diversity of EmsB profiles of *E. multilocularis* from red foxes and humans, with a particular focus on Asian variants. The profiles were hierarchically clustered into European and Asian units.

The results obtained in the current study were compared to those reported in^16^ to determine the continent of origin of the mitochondrial and nuclear genomic elements of each tapeworm. For the microsatellite profiling results used in this comparison, see Additional File 3 appended to^16^.

### Alignment-free approach

We introduce a novel computational approach: the 4D-Dynamic Representation of DNA/RNA Sequences combined with *K*-means clustering. The 4D-Dynamic Representation of DNA/RNA Sequences is an alignment-free bioinformatics method recently developed by us^32^. DNA or RNA sequences are transformed into sets of ‘material points’ in a 4D space, forming 4D-dynamic graphs. These graphs are treated as ‘rigid bodies’ and characterised by descriptors analogous to those used in classical dynamics, such as centres of mass and principal moments of inertia. For visualization, 2D and 3D projections of these graphs are employed^32,33^.

This bioinformatics method was integrated with *K*-means clustering, an unsupervised machine learning algorithm widely used in artificial intelligence. Previously, *K*-means clustering was combined with another of our bioinformatics approaches^34^. Here, we present cluster plots generated by applying *K*-means clustering to the descriptors derived from the 4D-Dynamic Representation of DNA/RNA Sequences.

## Results

### PCR amplification and sequencing of the complete *cox1* gene

We obtained complete coding sequences of the *cox1* gene for 252 out of 263 tapeworms from 59 red foxes. PCR was repeated for 11 samples but yielded no product.

### Population genetic analysis

**Fig. 2:**
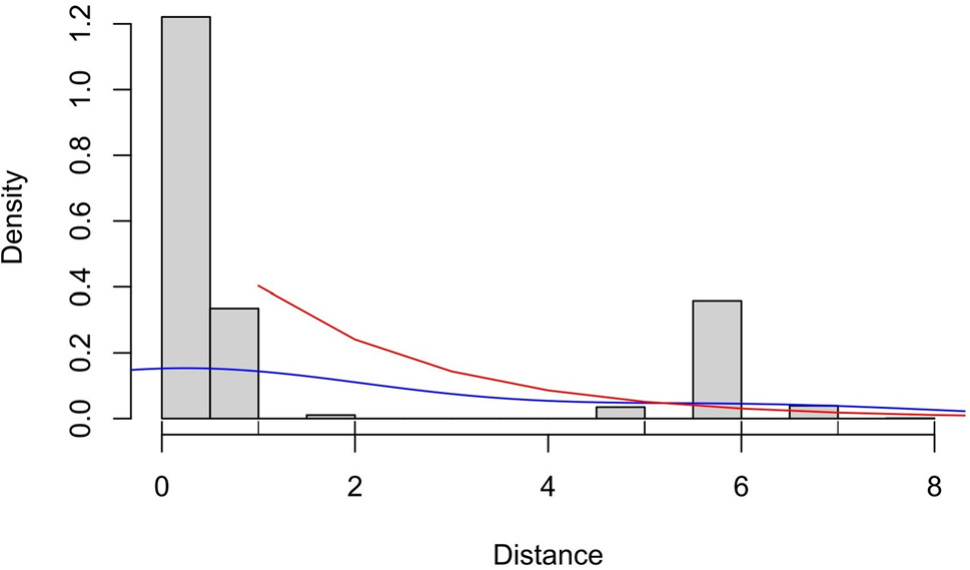
Observed distribution of pairwise distances between the 252 complete coding sequences of the cytochrome c oxidase subunit 1 gene (1,608 bp) (mismatch distribution). The blue line shows the empirical density estimate, and the red line shows the expected distribution under a stable population model^46^.

Nine segregating sites in total allowed us to identify six haplotypes, three of which were absent from previous studies (Table 1). The highest values of all genetic diversity estimators were observed in the districts of Warmińsko-Mazurskie Voivodship, with the most haplotypes detected in Kętrzyn District and the highest haplotype diversity in Bartoszyce District. The lowest overall genetic diversity was noted in Augustów District (Table 2). The mismatch distribution was bimodal (Figure 2). The value of the fixation index (F_ST_) between two haplogroups: European (n = 221, excluding haplotypes Hap_2 and Hap_6) and Asian (n = 31, composed of haplotypes Hap_2 and Hap_6), equalled 0.97057. The observed genetic differentiation between the two haplogroups was greater than expected by chance under panmixia, as assessed by permutation tests (α = 0.05, all P < 0.001).

**Table 1:** Haplotypes of the cytochrome c oxidase subunit 1 gene (1,608 bp) observed in Poland, from this and previous studies, with their frequencies in the PCR dataset from the present study. Conventional haplotype labels are given in parentheses to maintain consistency across reports^13,14^.

Haplotype	**Origin**	**Frequency [no.]**	**Frequency [%]**	**Reference**	**139**	**182**	**220**	**237**	**289**	**364**	**368**	**482**	**527**	**675**	**688**	**742**	**822**	**1432**	**1492**	**1514**
Hap_1 (EmPL_cox_A)	European	194/252	77	^13,14^	C	T	A	C	C	G	C	G	A	G	T	C	A	G	A	G
Hap_4 (EmPL_cox_A2)	European	4/252	2	This study	.	.	.	.	.	.	.	.	.	.	.	.	.	.	G	.
Hap_5 (EmPL_cox_A3)	European	4/252	2	This study	.	.	.	.	.	.	T	.	.	.	.	.	.	.	.	.
Hap_3 (EmPL_cox_B)	European	19/252	8	^13,14^	.	.	.	.	.	.	.	.	.	.	.	.	.	.	.	A
EmPL_cox_B2	European	Not detected	Not detected	^14^	.	.	G	.	.	.	.	.	.	.	.	.	.	.	.	A
EmPL_cox_C	European	Not detected	Not detected	^13^	.	G	.	.	.	.	.	.	.	.	.	.	.	.	.	A
EmPL_cox_D	European	Not detected	Not detected	^13^	.	.	.	.	.	.	.	A	.	.	.	.	.	.	.	A
Hap_2 (EmPL_cox_E)	Asian	29/252	12	^13^	.	.	.	T	T	.	.	.	.	T	C	.	G	.	.	A
EmPL_cox_E2*	Asian	Not detected	Not detected	^14^	.	.	.	T	T	.	.	.	.	T	C	.	G	A	.	A
Hap_6 (EmPL_cox_E3)	Asian	2/252	1	This study	.	.	.	T	T	.	.	.	.	T	C	A	G	.	.	A
EmPL_cox_F	European	Not detected	Not detected	^13,14^	.	.	.	.	.	T	.	.	.	.	.	.	.	.	.	A
EmPL_cox_G	European	Not detected	Not detected	^13,14^	.	.	.	.	.	.	.	.	G	.	.	.	.	.	.	A
EmPL_cox_G2	European	Not detected	Not detected	^14^	T	.	.	.	.	.	.	.	G	.	.	.	.	.	.	A

^*^The 1401-bp sequence EmPL_cox_E(pig), accession OQ874676, from^14^.

**Table 2:** Measures of *Echinococcus multilocularis* genetic variation in the cytochrome c oxidase subunit 1 locus (1,608 bp), calculated for the sampled districts.

**District(s)**	**Voivodship**	**No. of Sequences**	**S**	**h**	**H**_**d**_	**K**	**Pi**
Augustów	Podlaskie	45	0	1	0.00000	0.00000	0.00000
Bartoszyce	Warmińsko-Mazurskie	54	6	3	0.60098	2.45982	0.00153
Gołdap	Warmińsko-Mazurskie	53	7	4	0.44267	1.47750	0.00092
Kętrzyn	Warmińsko-Mazurskie	65	8	5	0.35481	1.27115	0.00079
Węgorzewo	Warmińsko-Mazurskie	18	6	2	0.42484	2.54902	0.00159
Puck, Wejherowo, Kartuzy, Kościerzyna	Pomorskie	17	1	2	0.22059	0.22059	0.00014
Total		252	9	6	0.38939	1.48103	0.00092

S – number of segregating sites, h – number of haplotypes, H_d_ – haplotype diversity, K – average number of nucleotide differences, Pi – nucleotide diversity.

**Fig. 3:**
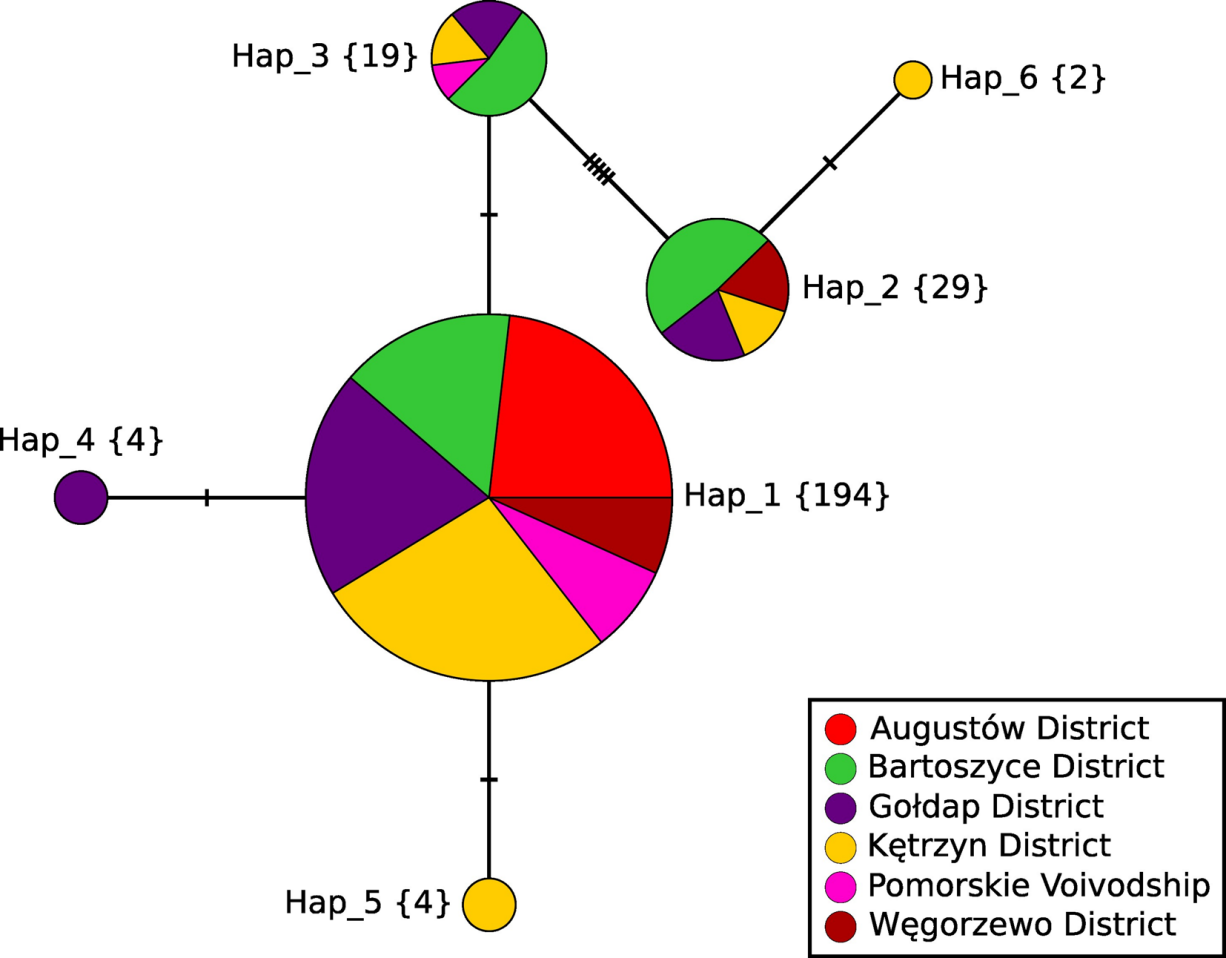
Median-joining haplotype network (ε = 0)^27^ generated from the PCR dataset of the 252 complete coding sequences of the cytochrome c oxidase subunit 1 gene (1,608 bp). Haplotypes were defined when at least one mutation occurred between two samples. The number of samples representing each haplotype is given in curly brackets. The number of mutations is shown as hatch marks.

Hap_1 was the most numerous and widespread haplotype (77%). It formed a starburst pattern (a central haplotype surrounded by derivatives) with Hap_3, Hap_4, and Hap_5. The second most-represented variant was Hap_2 (12%), present in all four districts of Warmińsko-Mazurskie Voivodship. Hap_2 and Hap_6 formed a satellite haplogroup separated from other haplotypes by five mutations (Figure 3).

**Fig. 4:**
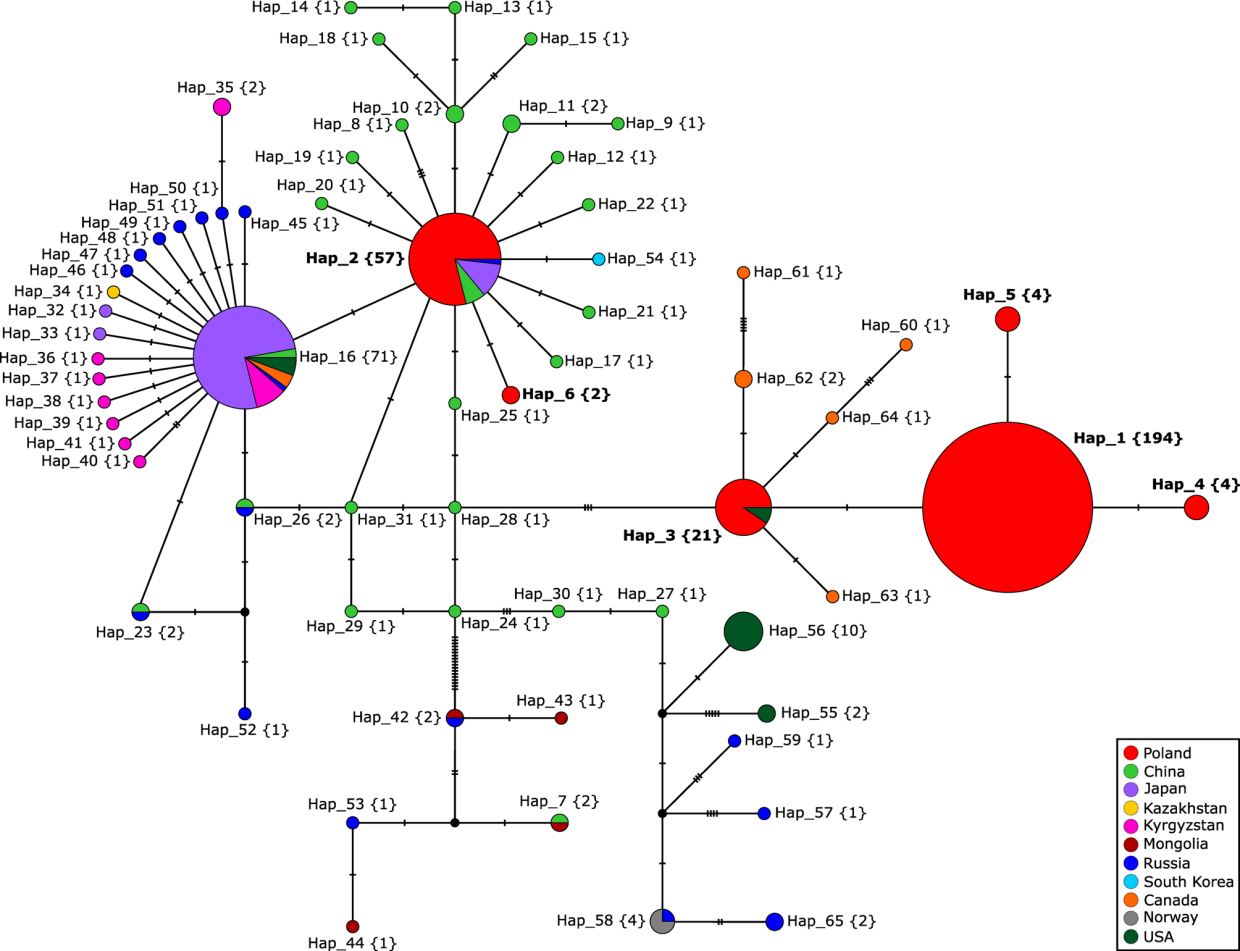
Median-joining haplotype network (ε = 0)^27^ generated from the PCR dataset of the 252 complete coding sequences of the cytochrome c oxidase subunit 1 gene (1,608 bp) combined with 146 records of Asian descent and 35 records from North America and the Arctic from GenBank (Supplementary File 1). Haplotypes were defined when at least one mutation occurred between two samples. The number of samples representing each haplotype is given in curly brackets. The number of mutations is shown as hatch marks. Haplotypes not sampled or extinct are represented by black circles.

In the intercontinental MJ haplotype network of a total of 433 sequences, Polish haplotypes blended into a reticulated pattern of Asian haplotypes represented by *E. multilocularis* from the Chinese province of Xinjiang (Figure 4), filling the five-mutation distance between Hap_2 and Hap_3 depicted in Figure 3. A Mongolian haplogroup was formed 18 mutations apart from the closest, Chinese (Xinjiang), haplotype. Some of these parasites originated from red and corsac foxes, a wolf, and a human from Hulunbuir (Inner Mongolia), Ulaanbaatar, and an unspecified area of Mongolia. Others came from Russian flat-headed voles collected on Olkhon Island on Lake Baikal (Irkutsk Oblast) and in the Altai Republic (Figure 4).

Haplogroups centred around Hap_16 and Hap_2 created a starburst pattern, with Hap_6 from two red foxes hunted in Kętrzyn District forming one of the rays of Hap_2. Hap_16 contained mainly Japanese, Russian, and Kyrgyz samples. Hap_2 comprised predominantly Polish samples—including tapeworms from previous studies^12,13^—as well as four Chinese samples (Qinghai, Sichuan, Xinjiang), seven Japanese adult tapeworms, and one Russian metacestode from the Altai region (Figure 4).

Sequences from Anabarsky and Nizhnekolymsky Districts (Russia, Yakutia), Svalbard (Norway), Alaska/St. Lawrence Island (USA)—regarded as Arctic regions, but also the states of South Dakota and Indiana (USA), were indirectly connected to haplotypes from Xinjiang (China), through missing haplotypes. Six Canadian haplotypes, from the provinces of British Columbia, Alberta, and Saskatchewan, formed rays stemming from the predominantly Polish Hap_3, which also included two samples from the state of Missouri. The remaining three samples from Canada and four samples from St. Lawrence Island belonged to Hap_16 (Figure 4).

### Timetree

**Fig. 5:**
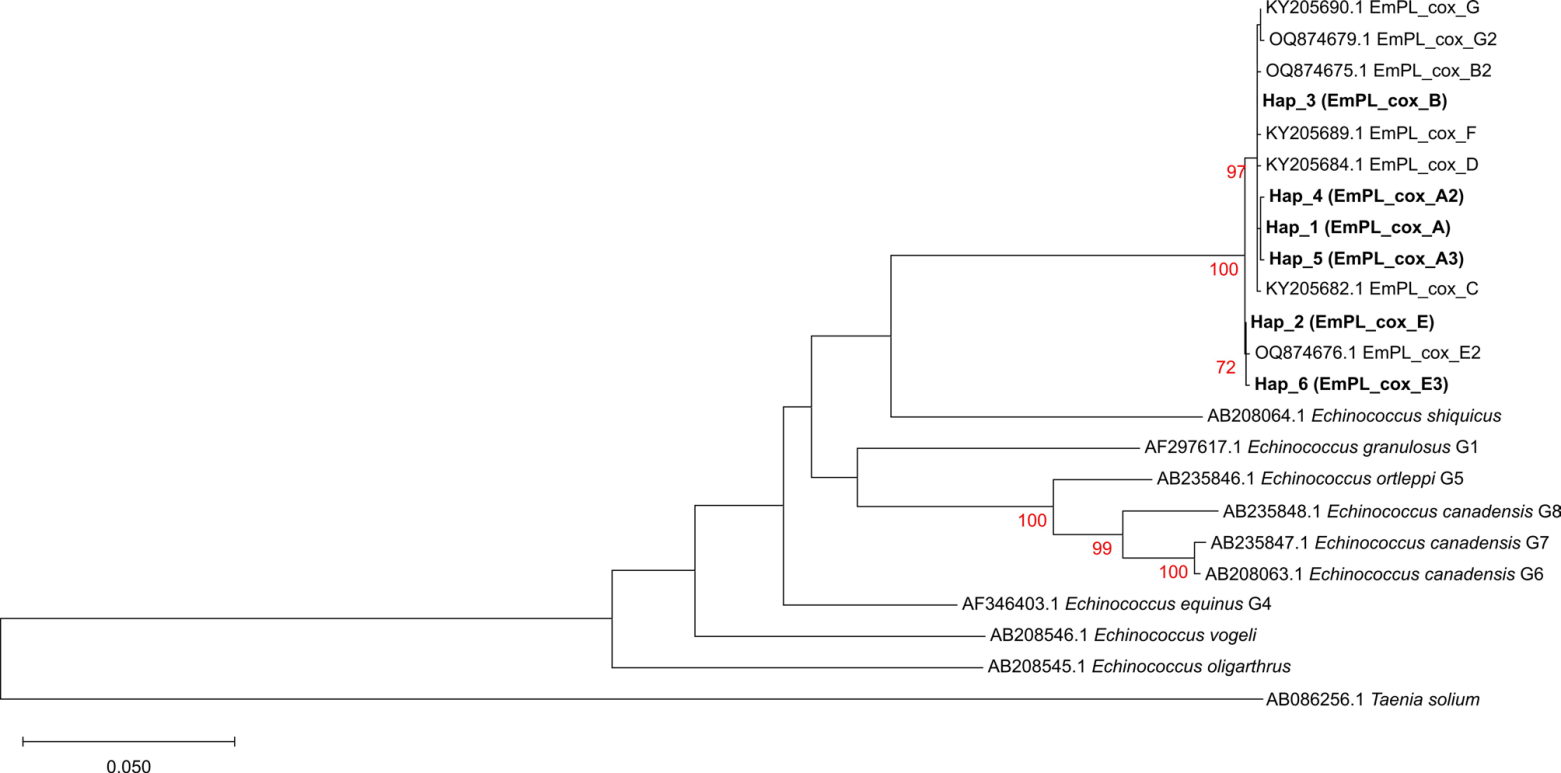
Phylogeny inferred using the Maximum Likelihood method and the Tamura-Nei model of nucleotide substitutions^47^. The tree with the highest log likelihood (-6,104.75) is shown. Bootstrap support values greater than 70% (1,000 replicates) are shown in red below the branches^48^. The initial tree for the heuristic search was selected based on superior log-likelihood between a Neighbour-Joining (NJ) tree^49^ and a Maximum Parsimony (MP) tree. The NJ tree was generated using a matrix of pairwise distances computed using the Tamura-Nei model^47^. The MP tree had the shortest length among 10 MP tree searches, each initiated with a randomly generated starting tree. Evolutionary rate variation among sites was modelled using a discrete Gamma distribution with 5 categories (+G, parameter = 0.8023), assuming that 50.02% of sites were evolutionarily invariant (+I). The analysis included 23 coding nucleotide sequences (total length 1,683 bp) and involved 1^st^, 2^nd^, and 3^rd^ codon positions, as well as non-coding positions. For details on haplotype labelling, see Table 1.

**Fig. 6:**
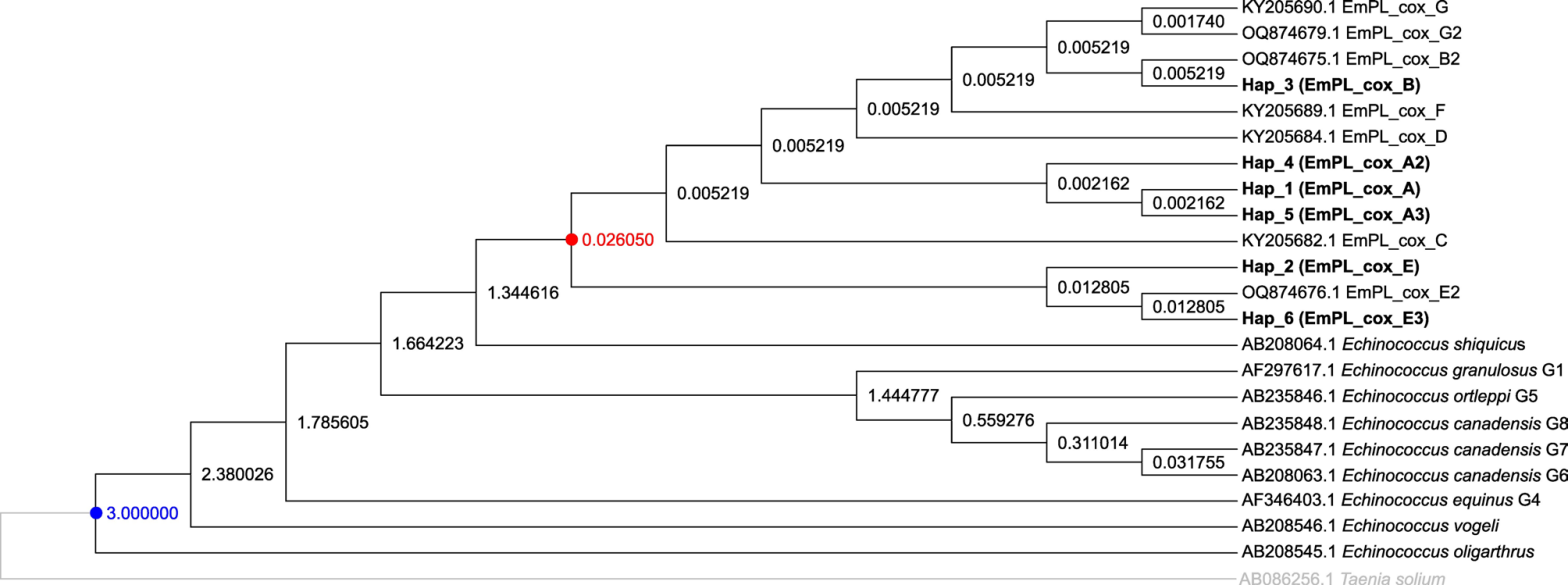
Timetree inferred by the RelTime method^50,51^ using the Tamura-Nei model of nucleotide substitutions^47^. Branch lengths were computed using the Maximum Likelihood method^52^. Evolutionary rate variation among sites was modelled using a discrete Gamma distribution across five categories (+G, parameter = 0.8023), assuming that 50.02% of sites were evolutionarily invariant (+I). The RelTime analysis incorporated a single calibration to derive minimum and maximum bounds for the calibrated node (marked in blue in the tree)^53^. Divergence time estimates in millions of years are shown next to nodes in the tree. Notably, times are not estimated for nodes in the outgroup because the RelTime method does not assume that evolutionary rates are the same for ingroup and outgroup lineages^51^. The divergence time of the European and Asian haplogroups is marked in red. The analysis included 23 coding nucleotide sequences (total length 1,683 bp) and involved 1^st^, 2^nd^, and 3^rd^ codon positions, as well as non-coding positions. For details on haplotype labelling, see Table 1.

European and Asian haplotypes formed distinct lineages in the ML tree (Figure 5). The divergence time of the European and Asian haplogroups was estimated to be 26,050 years ago (Figure 6).

### Comparison with nuclear data

Results per tapeworm and per red fox are presented in Supplementary File 2.

*Results per tapeworm*: Eighty per cent of the 252 tapeworms were of European origin, as indicated by the nuclear microsatellite marker EmsB and the mitochondrial marker *cox1*. Tapeworms of Asian descent constituted 6% of the total worm pool. Thirty-five parasites were of mixed origin (14%), i.e., exhibited different origin in mitochondrial and nuclear DNA. The proportion of Asian tapeworms in northern and northeastern Poland (15/252) was comparable to the proportion calculated for the whole country (13/349 from^12^) (Fisher’s exact test for count data, α = 0.05, P = 0.2402). However, the proportion of Euro-Asian hybrids was substantially higher in the study area (35/252) than at the national level (17/349 from^12^) (Fisher’s exact test for count data, α = 0.05, P = 0.0002).

*Results per red fox*: Five worms per fox were both genotyped and profiled for 41 red foxes. Twenty-three red foxes (56%) harboured European tapeworms with the most common EmsB pattern and haplotype, Pol-B/PH-2 and Hap_1, respectively. Two red foxes from Bartoszyce District contained only Asian tapeworms (5%). All five tapeworms from four red foxes—two from Gołdap District, one from Węgorzewo District, and one from Pomorskie Voivodship—were Euro-Asian hybrids (10%). Two red foxes from Bartoszyce District and two from Kętrzyn District were infected with European and hybrid tapeworms. One red fox from Gołdap District and one from Kętrzyn District were infected with European and Asian tapeworms. One red fox from Kętrzyn District contained tapeworms of European, Asian, and mixed origin.

### 4D-Dynamic representation of DNA/RNA Sequences combined with *K*-means clustering

Biological sequences are complex objects, making it uneasy to represent their structure and to define a similarity measure. The similarity space for such objects is multidimensional; only simple, one-dimensional entities can be classified uniquely using a single similarity metric. Complex objects may appear similar in one respect yet exhibit significant differences in another. Descriptors reflecting different aspects of similarity can be treated separately or combined into a single composite measure. A unified measure often results from either averaging across multiple aspects or disregarding most of them. Each bioinformatics method highlights distinct facets of similarity, and novel approaches continue to emerge. Standard alignment-based methods can overlook subtle, global patterns or topological distributions of the nucleobases within sequences. Our method is designed to quantify these higher-order properties, which are not directly accessible through standard similarity measures. Our goal is to uncover potential structural and organisational similarities that might be conserved even in the absence of strong sequence alignment.

**Fig. 7:**
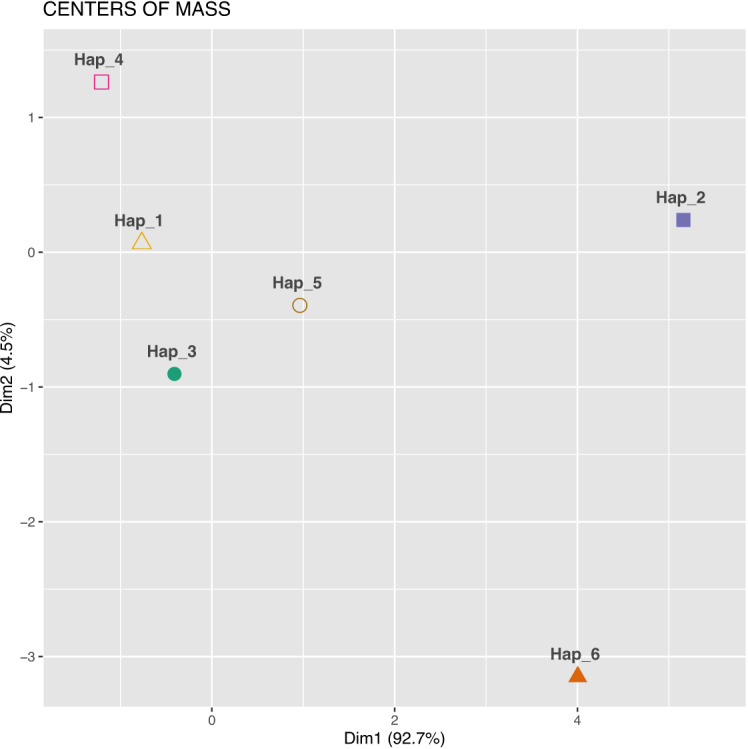
Cluster plot obtained using *K*-means clustering, with the centre of mass coordinates of the 4D-dynamic graphs as input data. (The computational framework for the 4D-dynamic method was developed by our team; the implementation and *K*-means clustering were performed in R^54^).

**Fig. 8:**
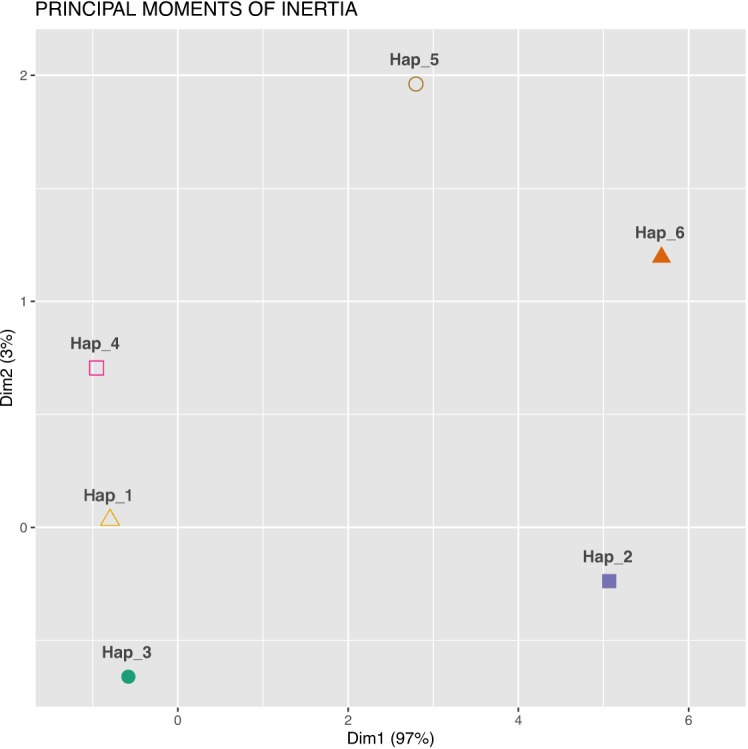
Cluster plot obtained using *K*-means clustering, with the principal moments of inertia tensor elements of the 4D-dynamic graphs as input data.

The relationships between sequences are visualised in a non-standard manner in Figures 6 and 7. Figure 7 displays a cluster plot generated using the coordinates of the centres of mass of the 4D-dynamic graphs as input for the *K*-means clustering algorithm. Figure 8 presents an analogous cluster plot based on the principal moments of inertia tensor elements of the graphs (see^32^ for mathematical details).

Both plots reveal six distinct point clusters. Sequence relationships can be analysed by comparing the Euclidean distances between these clusters. Notably, the distances between three point clusters—Hap_1, Hap_3, and Hap_4—are small in both visualisations.

The new method enables examination of different aspects of sequence similarity. For instance, Hap_5 exhibits varying distances from the three aforementioned clusters in the two plots, highlighting distinct relational patterns. To align with conventional bioinformatics approaches, the two descriptors—the centre of mass and the principal moment of inertia—can be combined into a single metric, as demonstrated in^34^, yielding an averaged similarity measure.

**Fig. 9:**
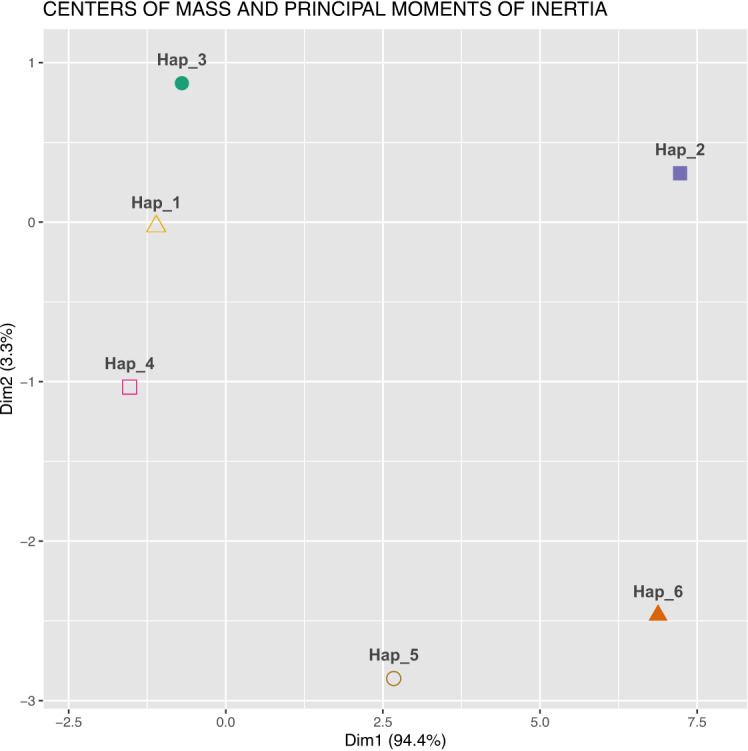
Cluster plot obtained using *K*-means clustering, with both the centres of mass coordinates and the principal moments of inertia tensor elements of the 4D-dynamic graphs as input data.

In the present work, to integrate the two descriptors, we extended the input dataset for the *K*-means clustering algorithm to include both the centres of mass coordinates and the principal moments of inertia tensor elements of the 4D-dynamic graphs representing the sequences. The resulting cluster plot is shown in Figure 9.

**Fig. 10:**
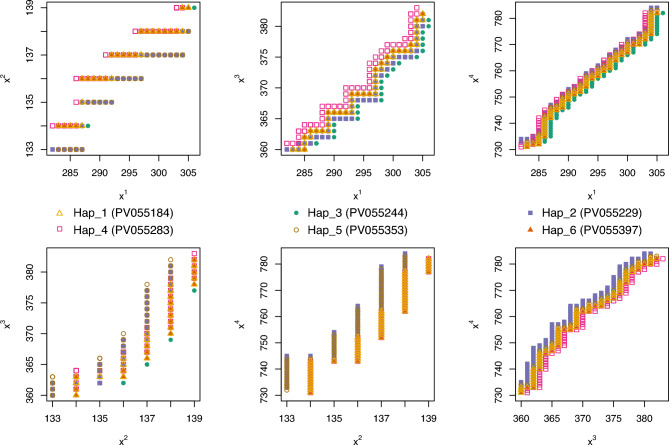
2D projections of the 4D-dynamic graphs representing fragments of the sequences.

Additionally, the method enables the visual inspection of sequence similarity and the identification of regions with significant or minimal variation. As the method operates in a multidimensional (4D) space, the results are graphically represented as two- or three-dimensional projections of the 4D-dynamic graphs. Since the analysed sequences are highly similar, their differences are difficult to discern in 3D projections. We have therefore computationally identified specific sequence fragments where these distinctions are most pronounced. These critical regions are shown as 2D projections of the 4D-dynamic graphs and presented in Figure 10.

## Discussion

In this study, we explored the *cox1* diversity of *E. multilocularis* from red foxes in northern and northeastern Poland, tested the hypothesis that the presence of Asian haplotypes in Poland is a consequence of their introduction from Northeast Asia to Eastern Europe, and compared the results with previously acquired EmsB data^16^. We used standard population genetic and phylogenetic tools and a novel approach to sequence analysis, the 4D-Dynamic Representation of DNA/RNA Sequences combined with *K*-means clustering.

The latter successfully demonstrated differences between the six observed haplotypes using physical descriptors of DNA sequences. Within this model, DNA sequences are represented as clouds of material points in a four-dimensional space (referred to as 4D-dynamic graphs), with each sequence yielding a unique spatial distribution. This abstract representation enables their description using formulas analogous to those of classical mechanics. To numerically characterise these 4D-dynamic graphs, we employ quantities equivalent to dynamical descriptors. In classical mechanics, common measures for describing the mass distribution of a rigid body include the centre of mass and the moment of inertia. The moment of inertia about a given axis quantifies how mass is distributed relative to that axis; the moments calculated about the principal axes are known as the principal moments of inertia. We extend this concept to describe distributions of other quantities: by assigning a conceptual ‘mass’ to each point in the 4D-dynamic graph, we define a graph moment of inertia analogous to its physical counterpart.

This method provides a fundamentally different and complementary framework for analysing sequence relationships. A key outcome of this analysis is that our approach independently confirms the major global patterns observed in networks constructed using standard methods. This mutual validation both strengthens the established results presented in this work and underscores the relevance of our novel descriptors.

A considerably larger sampling size per area than in other studies^12,13,35^ allowed us to detect rare haplotypes, such as Hap_4 (4/252, 2%), Hap_5 (4/252, 2%), and Hap_6 (2/252, 1%). It also elucidated the intestinal genetic diversity of *E. multilocularis* in local red foxes. We observed the highest worm diversity per individual fox near Kaliningrad Oblast, likely due to the prevalence of *E. multilocularis* in red foxes, ranging from 26.0% to 52.5% along the Polish-Russian border^18^. Small mammals being the main source of infection for canids, this finding raises the question of metacestode diversity and availability in prey in local transmission hotspots.

The proportion of Euro-Asian hybrids was higher in northern and northeastern Poland than at the national level. This difference could be attributed to a relatively high metacestode diversity in available prey, resulting in more frequent mixed infections among red foxes. Because wildlife does not respect administrative boundaries, the influence of adjacent Russian and Lithuanian populations needs to be considered to account for the observed genetic richness and hybrid frequency.

The median-joining haplotype network in Figure 3 revealed the demographic history of the investigated metapopulation. The starburst pattern—with the most-represented core haplotype Hap_1 and three less-numerous rays one mutation apart from Hap_1—is likely a result of the founder effect^3^. It typically emerges when pioneers, genetically less diverse than their ancestors, undergo sudden expansion^36^. Such tapeworms form the majority of *E. multilocularis* individuals in the investigated area.

The Hap_2/6 satellite in the network and a bimodal mismatch distribution (MMD) pattern (Figure 2) indicate secondary contact between two previously isolated metapopulations with distinct genetic lineages, namely European and Asian. Their interbreeding resulted in hybridisation detected in this and the previous study^12^. The MMD peaks at low genetic distances reflect intra-population variation stemming from relatively recent within-group mutations (Poland). The peaks at high distances are symptomatic of inter-group variation (Poland–Asia) accumulated over time, most likely due to geographical distance. The time of bifurcation into the European and Asian *E. multilocularis* estimated in this study roughly corresponds to the range of 37,000–60,000 years estimated by a rapid molecular clock of 10% divergence per million years^19^. It is more similar to the 30,800 years calculated by^37^; our estimate is closer to, but still predates, the Last Glacial Maximum, which took place approximately 20,000 years ago. The fixation index supports this interpretation, as the genetic separation between the European and Asian haplogroups is almost complete (F_ST_ ≈ 1).

Unfortunately, a shortage of complete *cox1* sequences from Asia, especially Russia—the largest country on the Asian continent (by area)—prevents a conclusive identification of the geographic whereabouts of the source population(s) of haplotypes Hap_2 and Hap_6. The Japanese samples with *cox1* identical to Hap_2 were collected in the sub-prefectures of Kushiro and Nemuro, eastern Hokkaido^38^. In the original mitogenomic study, only these samples—a subset of a Japanese dataset—grouped with Sichuan haplotypes, implying historical animal transport from mainland Asia to Hokkaido.

It seems plausible that Asian variants of *E. multilocularis* were introduced into northeastern Poland either by raccoon dogs migrating mainly from the Baltic countries and Belarus after multiple anthropogenic introductions of the species into Eastern Europe^5^, or by other wild canids after earlier deposition of Asian variants further east by raccoon dogs. Current European raccoon dog populations are descendants of southeastern Siberian individuals of the subspecies *Nyctereutes procyonoides ussuriensis* Matschie, 1907, transported to European parts of the Soviet Union between the late 1920s and the late 1950s^39,40^. Their predecessors inhabited mainly the Amur and Ussuri river valleys, the shores of the Sea of Japan, and eastern China. Initially bred for their thick fur, the animals were released into the wild. They spread rapidly, and in 1955, after crossing the Polish-Belarusian border, a raccoon dog was spotted in Narewka Forest District, in what is now Podlaskie Voivodship. The same year, another individual was observed in Strzelce Forest District, over the Polish-Ukrainian border, in modern-day Lubelskie Voivodship. It took raccoon dogs little time to enter inland and reach as far as East Germany, where they were first spotted in the early 1960s^40^.

If this explanation is correct, introduced *E. multilocularis* lineages still closely resemble their ancestral populations in Asia. Tapeworm clustering in phenograms and networks appears to support this view in light of historical records but with limitations. Formally, the available Chinese reference sequences come from West-Central China, and we lack genetic information from Northeast Asia. Still, the general undersampling of the Asian continent leaves us in the dark about the geographical range of Hap_2 and Hap_6, both of which may be widespread enough to be present in Northeast Asia.

Human-mediated introduction of *E. multilocularis* is not unprecedented. Major and minor haplogroups in Hokkaido likely result from animal introductions from St. Lawrence Island and Sichuan, respectively, as supported by historical evidence and the aforementioned mitogenomic study^38^. Similarly, multiple events of transportation of red foxes and/or domestic dogs from continental Europe are the most likely reason for the invasion of European genotypes into Canada^41,42^ and the USA^43–45^, as reflected by the composition and configuration of Hap_3 and Hap_60–64 in Figure 4. With more regional research like ours in the future, preferably based on whole mitogenomes, and insights from historical records, it may be possible to trace geographically aberrant lineages back to their source and explain their journey.

## Supplementary Information

Supplementary Information 1.

Supplementary Information 2.

## Data Availability

The 252 complete coding sequences of the cytochrome c oxidase subunit 1 gene (1,608 bp) obtained in this study were submitted to GenBank and received accessions PV055184–PV055435.
